# Spectrum of congenital anomalies of the kidney and urinary tract in a pediatric nephrology clinic in Ethiopia and factors associated with advanced chronic kidney disease: A cross-sectional study

**DOI:** 10.1371/journal.pgph.0003869

**Published:** 2024-11-25

**Authors:** Biniam Endale Geleta, Bezaye Abebe Bekele, Mekbeb Afework

**Affiliations:** 1 Department of Anatomy, School of Medicine, Addis Ababa University, Addis Ababa, Ethiopia; 2 Department of Child Health, School of Medicine, Addis Ababa University, Addis Ababa, Ethiopia; University of Alberta Faculty of Medicine & Dentistry, CANADA

## Abstract

Chronic kidney disease (CKD) is a significant health concern for children with congenital anomalies of the kidney and urinary tract (CAKUT). To address the limited data on CKD prevalence and risk factors in Ethiopia, this study focuses on a specific cohort of CAKUT patients at the pediatric renal unit of Tikur Anbessa specialized hospital. We aim to determine the prevalence of CKD in Ethiopian CAKUT patients, identify independent predictors of advanced stage CKD, and explore the distribution of CKD among different CAKUT phenotypes. Medical charts of children with CAKUT who visited pediatric renal unit were reviewed. Logistic regression analyses were performed to determine the independent predictors of advanced CKD. Statistical significance was set at p ≤ 0.05.The study included 129 male subjects (70.9%). Only 25.3% of CAKUT cases were diagnosed prenatally. About 52.2% of patients had unilateral CAKUT. Posterior urethral valves (PUV) were the most commonly diagnosed CAKUT phenotypes (27.5%). Fifty-three (29.1%) of the patients had CKD, and stage 3 was the most diagnosed CKD (37.7%). CKD development was observed more frequently in patients with isolated PUV (39.6%). Factors like low birthweight, prematurity at birth, and the presence of bilateral CAKUT emerged as independent risk factors for developing advanced stage CKD. This study reveals a significant burden of CKD in children and adolescents with CAKUT. Despite its importance, prenatal CAKUT detection remains low. Posterior urethral valves are the most prevalent phenotype, associated with a higher risk of CKD. The research also highlights independent predictors for advanced CKD development.

## 1. Introduction

The burden of chronic kidney disease (CKD) is increasing worldwide. Congenital Anomalies of the kidney and urinary tract (CAKUT) are a major culprit behind CKD in children [1,2].

Congenital anomalies of the kidney and urinary tract is one of the most common types of birth anomalies, accounting for 20% to 50% of all anomalies at birth and one third of prenatal ultrasound abnormalities [3–5]. Its global prevalence is ranging from 3–13 per 10,000 live births [6].

Previous studies found a strong link between CAKUT and CKD, with CAKUT contributing to 44–59% of CKD cases in developed countries [7–9]. While African data is more limited, studies from Sudan, Egypt, South Africa, and Nigeria report varying rates of CAKUT related CKD, ranging from 17.5% to 46% [10–15].

Congenital anomalies of the kidneys and urinary tract present diverse phenotypes. The development of CKD varies among these phenotypes. Specific subgroups cause CKD in a noticeably short course of time, whereas other subgroups are known to progress slowly or not progress at all [3,8,16–21].

While CKD is a significant concern for children with CAKUT, data on its prevalence and risk factors in this population, particularly in Ethiopia, is limited. This study aims to bridge this knowledge gap by investigating the prevalence of CKD in a specific cohort of Ethiopian CAKUT patients. Additionally, the study will identify independent predictors of advanced stage CKD and explore the distribution of CKD among different CAKUT phenotypes to determine if certain anomalies are associated with a higher risk of CKD.

Addressing these questions will provide valuable insights into improving the early detection, management, and prognosis of CKD in Ethiopian children with CAKUT. Understanding the disease burden will inform resource allocation for targeted prevention, early detection, and treatment programs within the Ethiopian healthcare system. Ultimately, this research has the potential to not only bridge the knowledge gap regarding CKD in CAKUT but also to translate into tangible improvements in the quality of care for these vulnerable patients.

## 2. Methods

### 2.1 Study design and setting

This was a cross-sectional study. All patients diagnosed with CAKUT in the Tikur Anbessa specialized hospital (TASH) pediatric renal unit between September 2020 and August 2022 were included.

### 2.2 Data collection

In this retrospective study, patient charts were reviewed between February 10th and 11th, 2023. All data were anonymized before access for research purposes. A unique coding system replaced patient identifiers, ensuring researchers could not identify individuals during data collection and analysis.

Biographic data, type of CAKUT diagnosis, age at diagnosis, method of diagnosis, and radiographic examination results were recorded. Potential risk factors associated with CKD development including history of low birthweight, prematurity at birth, time of CAKUT diagnosis, CAKUT bilaterality, and presence of multiple CAKUT were also recorded. The CAKUT diagnosis subgroups of PUV, VUR, multi-cystic dysplastic kidney (MCDK), renal agenesis/solitary kidney, ectopic kidneys, horseshoe kidneys, hypoplastic kidneys, pelvi-ureteric junction obstruction (PUJO), vesico-ureteral junction obstruction (VUJO), bladder exstrophy, and duplicated excretory systems were included in the analysis. These CAKUT cases were diagnosed following a comprehensive clinical evaluation and the application of appropriate imaging diagnostic methods. These methods included abdominopelvic ultrasound, cystoscopy, voiding cystourethrography, and intravenous pyelogram, selected based on the suspected type of CAKUT.

To handle missing data, we adopted a practical strategy that involved both exclusion and imputation. We excluded variables with a significant proportion of missing values, and for categorical variables with a limited number of missing values, we employed frequent category imputation method.

Chronic kidney disease diagnoses and stages were obtained from patients’ medical records. These records employed the bedside modified Schwartz formula (eGFR = 0.413* ht (cm) / serum creatinine (mg/dl)), a standard practice at this healthcare facility to calculate eGFR values [22]. Serum creatinine levels were determined using the Jaffe method. Based on these eGFR values, CKD stages were categorized as follows: Stage 1 (> 90 ml/min/1.73 m^2^), Stage 2 (60–89 ml/min/1.73 m^2^), Stage 3 (30–59 ml/min/1.73 m^2^), Stage 4 (15–29 ml/min/1.73 m^2^), and Stage 5 (< 15 ml/min/1.73 m^2^). Notably, as per the established criteria, a diagnosis of CKD stages 1 and 2 (eGFR > 60 mL/min/1.73 m^2^) necessitates the presence of at least one of additional markers of kidney damage (albuminuria > 30 mg/day, urine sediment abnormalities, electrolyte and other abnormalities due to tubular disorders, histological abnormalities, structural abnormalities detected by imaging and history of renal transplantation) [23].

For the purposes of this study, advanced CKD is operationally defined as an eGFR less than 60 ml/min/1.73 m^2^.

### 2.3 Statistical analysis

The analysis was performed using the IBM SPSS software version 24. Multiple responses were considered in the analysis of the CAKUT phenotypes. Logistic regression analysis was used to investigate potential factors for CKD development.

The CAKUT phenotypes and potential risk factors including premature birth, low birthweight, presence of, CAKUT bilaterality, presence of multiple CAKUT, time of CAKUT diagnosis and sex were considered in the univariate analysis. The risk factors with p-values less than or equal to 0.05 were selected as candidate variables for multivariate logistic regression analyses. The Hosmer-Lemeshow test was used to assess the goodness-of-fit of the model. Statistical significance was set at p ≤ 0.05.

## 3. Results

One hundred eighty-two charts of children with CAKUT were reviewed. Of these, 129 (70.9%) were male, and only 46 (25.3%) were diagnosed prenatally. The remaining 136 (74.7%) were diagnosed postnatally, often following urinary tract infections (79.4%). Ninety-five cases (52.2%) involved unilateral CAKUT. The median age at diagnosis was 48 months, with an IQR of 73 months (11 months to 84 months) (S1 Data).

The CAKUT diagnoses were categorized into eleven phenotypes. The PUV phenotype was the most prevalent among study participants, with 50 individuals (27.5%) affected. PUJO was the second most common phenotype, observed in 38 cases (20.9%). Multiple CAKUT were identified in 27 (14.8%) of the children, with the most common combinations being PUJO and VUR in six cases, and MCDK and PUJO in three cases. Three subjects were diagnosed with triple CAKUT phenotypes (Table 1).

**Table 1:** Distribution of CAKUT phenotypes among study subjects.

CAKUT phenotypes diagnosed	Frequency	Percentage
Posterior urethral valves	47	25.8
Ectopic kidney	28	15.4
Multi cystic dysplastic kidney	24	13.2
Pelvi-ureteric junction obstruction	23	12.6
Solitary kidney	15	8.2
Hypoplastic kidney	7	3.9
Vesico ureteral junction obstruction	5	2.8
Horseshoe kidney	4	2.2
Bladder exstrophy	1	0.6
Primary vesico-ureteric reflux	1	0.6
Pelvi-ureteric junction obstruction + Vesico-ureteric reflux	6	3.3
Pelvi-ureteric junction obstruction + Multi cystic dysplastic kidney	3	1.7
Multi cystic dysplastic kidney + Vesico-ureteric reflux	2	1.1
Posterior urethral valves + Multi cystic dysplastic kidney	2	1.1
Solitary kidney + Vesico-ureteric reflux	2	1.1
Pelvi-ureteric junction obstruction + Ectopic kidney + Vesico-ureteric reflux	1	0.6
Pelvi-ureteric junction obstruction + Solitary kidney + Vesico-ureteric reflux	1	0.6
Pelvi-ureteric junction obstruction + Multi cystic dysplastic kidney + Vesico-ureteric reflux	1	0.6
Other co-occurrences*	9	4.9
Total	182	100

**Note**: **CAKUT**: Congenital anomalies of the kidney and urinary tract.

* Include one case for each of: PUV & Hypoplastic kidney; Ectopic kidney & Horseshoe kidney; PUJO & Horseshoe kidney; Ectopic kidney & VUR; VUJO & VUR; PUJO & Hypoplastic kidney; PUJO & Solitary kidney; MCDK & Horseshoe kidney; Duplex collecting system & VUR.

Of the 182 study subjects with CAKUT, 53 (29.1%) had CKD. Of these 182 subjects, 20 (11%) had Stage 3 CKD and 6 (3.3%) had end-stage kidney disease (ESKD). Advanced CKD accounted for 34 (64.2%) of all CKD cases.

Regarding CKD development, subjects diagnosed with PUV were more likely to develop CKD. Of the 53 subjects with CKD, 21 (39.6%) had isolated PUV, while 6 (11.3%) had isolated ectopic kidney, and 5 (9.4%) had isolated PUJO. Additionally, advanced stage CKD was more common in subjects with PUV. Of the 34 subjects with advanced CKD, 15 (44.1%) had isolated PUV. Importantly, among patients with PUV who developed advanced CKD, only one had an additional CAKUT condition (Table 2).

**Table 2:** Distribution of different CKD stages among various CAKUT phenotypes.

CAKUT phenotypes	Stages of CKD			Total
	Stage 1 CKD	Stage 2 CKD	Advanced stage CKD	
Posterior urethral valves	1	5	15	21
Ectopic kidney	2	3	1	6
Pelvi-ureteric junction obstruction	1	0	4	5
Multi cystic dysplastic kidney	0	2	1	3
Vesico-ureteral junction obstruction	0	0	3	3
Pelvi-ureteric junction obstruction + Vesico-ureteric reflux	1	0	2	3
Hypoplastic kidney	0	0	2	2
Others*	1	3	6	10
Total	6	13	34	53

**Note**: **CAKUT**: Congenital anomalies of the kidney and urinary tract **CKD**: Chronic kidney disease.

^*:^ **Include:** One case of CKD stage 1 in PUJO + MCDK.

One case of CKD stage 2 each in Bladder exstrophy; Solitary kidney; PUV + Hypoplastic kidney.

One case of advanced stage CKD each in Ectopic kidney + Horseshoe kidney; PUJO + Horseshoe kidney.

PUV + MCDK; Ectopic kidney + VUR; VUJO + VUR; PUJO + Solitary kidney.

About 65.4% of patients with premature birth; 54.3% of those with history of low birthweight; 40.7% of patients with multiple CAKUT; and 43.7% of patients with bilateral CAKUT had CKD.

In the univariate analysis, the diagnosis of PUV, MCDK, VUJO, VUR and presence of factors such as CAKUT bilaterality, history of low birthweight, and prematurity at birth, were found statistically associated with the development of advanced stage CKD (p <0.05) (Table 3).

**Table 3:** Univariate and Multivariate logistic regression analysis of predictive factors for development of advanced stage CKD.

Predictive factors	Univariate analysis				Multivariate analysis			
	OR	p-value	95% C.I. for OR		OR	p-value	95% C.I. for OR	
			Lower	Upper			Lower	Upper
CAKUT bilaterality	7.0	< .001	2.8	18	5.0	0.015*	1.4	18.4
Low birthweight	6.0	< .001	2.6	13.8	3.8	0.013*	1.3	10.9
						
Prematurity at birth	6.4	< .001	2.6	15.8	4.9	0.006*	1.5	15.9

Posterior urethral valves	3.0	0.006	1.4	6.5				
Multi-cystic dysplastic kidney	8.7	0.036	1.2	66.5				
Vesico-ureteral junction obstruction	9.7	0.010	1.7	55.6				
Vesico-ureteric reflux	3.2	0.014	1.3	8.1				

**Note**: * Statistically significant: p≤ 0.05.

**CAKUT**: Congenital anomalies of the kidney and urinary tract **OR**: Odds ratio **CI**: Confidence interval

In the multivariate logistic regression, low birthweight [OR 3.8; 95% CI 1.3–10.9, p = 0.013], prematurity at birth [OR 4.9; 95% CI 1.5–15.9, p = 0.006], and presence of CAKUT bilaterality [OR 5; 95% CI 1.4–18.4, p = 0.015] were found to be independent predictors for development of advanced stage CKD (Table 3).

## 4. Discussion

Globally, CAKUT are major causes of pediatric CKD, accounting for 50–60% of cases. In the North American pediatrics renal transplant cooperative study (NAPRTCs) data, CAKUT were the most common cause of kidney failure requiring replacement therapy, with a prevalence of up to 56.2% [8,24–26]. There are different CAKUT phenotypes with different pathophysiology and clinical courses of CKD development [21].

This study investigated the burden of CKD in children with CAKUT. The majority of participants were male, with a male-to-female ratio of 2.4:1. This finding aligns with previous research by Anigilaje and Adesina (2019) and Okoronkw et al. (2020). This prevalence of males might be attributed to PUV, the most common CAKUT phenotype, which only affect males.

Early detection of CAKUT is crucial for mitigating the impact of related complications. Rosendahl (1990) [27] reported prenatal diagnosis rates of 60–85% in Finland. However, studies in Nigerian and Egyptian university hospitals found lower rates of 12.1% and 21.3%, respectively [18,28]. Of the CAKUT cases in our study, only 25.3% were diagnosed prenatally using antenatal ultrasound, similar to Egyptian findings [28]. The majority, 74.7%, were diagnosed postnatally, often following urinary tract infections (79.4%). This lower prenatal diagnosis rate in Africa may be due to economic factors that affect the availability and quality of healthcare services. Therefore, improving antenatal care services with ultrasound examinations is crucial in Africa, as prenatal and early postnatal diagnosis and management can help prevent or delay advanced CKD by preventing urinary tract infections.

Previous studies at TASH and other institutions have demonstrated a significant association between CAKUT and pediatric renal admissions, with rates ranging from 26.8% to 60% [8,29]. Our study found that 29.1% of children diagnosed with CAKUT also had CKD, which is lower than the prevalence reported in developed nations like those in Europe, North America, Spain, and Germany (48–59%) [7–9]. This disparity may be due to variations in factors such as the duration of patient follow-up, healthcare-seeking behaviors, availability of specialized pediatric urology teams, and the diagnostic resources available across different regions.

The pattern of CAKUT phenotypes vary across geographic regions. Studies from South Africa (44.8%), Nigeria (42.4%), and Egypt (36.4%) showed PUV as the most frequently diagnosed CAKUT phenotype [18,20]. Similarly, among the eleven CAKUT phenotypes identified in the present study, PUV were the most commonly diagnosed, with 27.5% of all CAKUT cases.

Several studies have linked CAKUT bilaterality to CKD development. Ibarra et al. (2022) found that the majority of CAKUT cases were unilateral (80.7%), consistent with our study’s findings of unilateral CAKUT in 52.2% of cases.

Research suggests that different CAKUT phenotypes can influence the course of CKD development [8,21]. Cetinkaya et al. (2020) identified PUV as a leading cause of CKD, accounting for 55% of cases. Our study aligns with these findings, with PUV representing the most prevalent CAKUT phenotype associated with advanced CKD (44.1%). This association might be explained by the anatomical location of PUV, which can obstruct the bladder outlet and contribute to bilateral urine reflux. While the overrepresentation of PUV in our cohort may be influenced by selection bias, the development of advanced CKD in boys with PUV may be further influenced by the associated hypodysplasia of the kidneys and the morphological and functional changes that the bladder undergoes.

While hypoplastic kidney has been identified as a risk factor for CKD in some populations [24], our study did not find a significant association between hypoplasia and CKD development. This may be due to the relatively small number of cases with hypoplasia in our sample or the fact that hypoplasia was not frequently observed in conjunction with other CAKUT anomalies in our study.

Beyond CAKUT phenotypes, several other factors influence CKD development in children with these anomalies. Studies have identified statistically significant associations between CKD and CAKUT bilaterality, male sex, premature birth, oligohydramnios, and presence of proteinuria upon admission [3,4,21,28,30]. Expanding upon these findings, our study confirms that low birthweight, prematurity, and presence of bilateral CAKUT emerged as independent risk factors for developing advanced stage CKD. These associations may be explained by the fewer and smaller glomeruli often associated with preterm birth and low birthweight. Additionally, the presence of bilateral CAKUT, can increase the burden on both kidneys and contribute to a higher risk of advanced CKD progression [24,31].

This study has made significant contributions to understanding CKD prevalence and risk factors among CAKUT patients in a specialized hospital pediatric renal unit in Ethiopia, a previously understudied area. By focusing on this high-risk population, the clinical relevance of our findings is enhanced. Through logistic regression analysis, we identified independent predictors of advanced CKD development, informing the development of early detection and intervention strategies.

However, this study has limitations inherent to retrospective data analysis. The exclusion of variables with extensive missing data and the use of imputation for categorical variables may introduce potential biases into our findings. Although these strategies are commonly used to handle missing data, they can distort variable distributions. Additionally, information on established CKD predictors, such as proteinuria, blood pressure, urinary tract infection frequency, and timing of surgical intervention, was not available for analysis. The absence of this data could potentially influence the study’s conclusions regarding the full spectrum of risk factors associated with CKD development in CAKUT patients.

Additionally, the generalizability of the findings to the entire Ethiopian CAKUT population might be limited due to selection bias, a common challenge in retrospective studies based on a single institution. Future prospective studies with a broader sample size could address these limitations and provide a more comprehensive understanding of CKD in CAKUT patients within the Ethiopian context.

## 5. Conclusion

This study highlights the significant burden of CKD among children with CAKUT at TASH pediatric renal unit in Ethiopia. Despite the importance of early detection, only 25% of CAKUT cases were diagnosed prenatally, emphasizing the need for improved access to prenatal ultrasound and healthcare services. Posterior urethral valves emerged as the most prevalent phenotype, associated with a higher risk of CKD compared to other phenotypes, underscoring the importance of phenotype identification for tailored treatment. Additionally, this study identified independent predictors of advanced CKD development.

## Supporting information

S1 Data All data of the study. (SAV)
